# MBP-binding DARPins facilitate the crystallization of an MBP fusion protein

**DOI:** 10.1107/S2053230X18009901

**Published:** 2018-08-29

**Authors:** Rajesh Gumpena, George T. Lountos, David S. Waugh

**Affiliations:** a Macromolecular Crystallography Laboratory, Center for Cancer Research, National Cancer Institute at Frederick, Frederick, MD 21702, USA; b Macromolecular Crystallography Laboratory, Basic Science Program, Leidos Biomedical Research Inc., Frederick National Laboratory for Cancer Research Sponsored by the National Cancer Institute, Frederick, MD 21702, USA

**Keywords:** crystallization chaperones, designed ankyrin-repeat proteins, dual-specificity phosphatase 1, maltose-binding protein, surface-entropy-reduction mutagenesis

## Abstract

The production of high-quality crystals is the main bottleneck in determining the structures of proteins using X-ray crystallography. In addition to being recognized as a very effective solubility-enhancing fusion partner, *Escherichia coli* maltose-binding protein (MBP) has also been successfully employed as a ‘fixed-arm’ crystallization chaperone in more than 100 cases. Here, it is reported that designed ankyrin-repeat proteins (DARPins) that bind with high affinity to MBP can promote the crystallization of an MBP fusion protein when the fusion protein alone fails to produce diffraction-quality crystals. As a proof of principle, three different co-crystal structures of MBP fused to the catalytic domain of human dual-specificity phosphatase 1 in complex with DARPins are reported.

## Abstract 2

Designed ankyrin-repeat proteins (DARPins) that bind to maltose-binding protein (MBP) with high affinity can facilitate the crystallization of an MBP fusion protein. The use of MBP-specific DARPins increases the probability of obtaining crystals.

## 1. Introduction   

X-ray crystallography continues to be an invaluable tool for determining protein structures at high resolution. X-ray structure determination has become extremely automated and fast owing to continuous technical improvements in molecular biology, protein production and computational tools (Abola *et al.*, 2000 ▸). However, the major impediment to this technique remains the production of high-quality diffracting crystals. To overcome this problem, a variety of ‘rescue’ strategies such as single-point mutations, the deletion of disordered regions by limited proteolysis, surface-entropy-reduction (SER) mutagenesis, mutagenesis to remove posttranslational modifications, the generation of individual domains out of larger proteins, crystallization with binding partners (or ligands), reductive methylation and crystallization of fusion proteins have been employed in efforts to obtain crystals of recalcitrant proteins (Dale *et al.*, 2003 ▸; Ruggiero *et al.*, 2012 ▸; Cooper *et al.*, 2007 ▸; Bell *et al.*, 2013 ▸; Longenecker *et al.*, 2001 ▸). However, owing to the stochastic nature of crystallization experiments, there remains a persistent demand for technological advances in the field of protein crystallization.

*Escherichia coli* maltose-binding protein (MBP) is frequently used as a ‘fixed-arm’ crystallization chaperone, and crystal structures of many MBP fusion proteins can be found in the Protein Data Bank (PDB) (Waugh, 2016 ▸). The differing conformations of MBP in the presence and absence of maltose further expand the crystallization space that can be sampled with MBP fusion proteins. Additionally, surface-entropy-reduction mutations have been introduced into MBP to facilitate the crystallization of MBP fusion proteins (Moon *et al.*, 2010 ▸; Jin *et al.*, 2017 ▸). Even so, like other rescue strategies, efforts to crystallize MBP fusion proteins fail more often than not.

Dual-specificity phosphatase 1 (DUSP1), also known as MAP kinase phosphatase 1 (MKP-1), is composed of a kinase-binding domain (KBD) and a catalytic domain (CD). Although it was the first human DUSP to be identified (Slack *et al.*, 2001 ▸), until recently the structure of neither its KBD nor its CD had been determined. Extensive efforts on our part to crystallize the DUSP1 CD alone or as a fusion protein with MBP were unsuccessful. However, well diffracting crystals of the MBP-DUSP1 CD fusion protein in complex with the monobody YSX1 (Gilbreth *et al.*, 2008 ▸) that binds with high affinity to MBP were readily obtained (Gumpena *et al.*, 2018 ▸). Designed ankyrin-repeat proteins (DARPins) that bind with high affinity to MBP have also been described (Binz *et al.*, 2004 ▸). To investigate whether these MBP-specific DARPins can also help to promote the crystallization of MBP fusion proteins, we tested the two MBP-specific DARPins off7 and mbp3_16 (DARPin 16) in concert with MBP fused to cata­lytically inactive DUSP1 CD (Cys258Ser). DARPin off7 has three (N3C) and DARPin 16 has two (N2C) designed ankyrin repeats inserted between the same N- and C-terminal capping repeats. Each designed repeat contains randomized residues that interact with MBP along with defined and random framework residues. Although DARPin off7 has previously been crystallized in complex with MBP, until now no information has been available on how DARPin 16 binds to this target.

## 2. Experimental   

### 2.1. Cloning, expression and purification   

Construction of the MBP-DUSP1 CD (Cys258Ser) fusion protein has been described previously (Gumpena *et al.*, 2018 ▸). Plasmids encoding DARPin off7 and DARPin 16 were a gift from Professor Andreas Plückthun’s laboratory (Binz *et al.*, 2004 ▸). The open reading frames encoding the DARPins with N-terminal His_6_ tags were amplified by polymerase chain reaction (PCR) with primers PE-2888 and PE-2889 (Table 1 ▸). The resulting PCR amplicons were digested with NdeI and XhoI (New England BioLabs, Ipswich, Massachusetts, USA) and inserted between these sites in the pET-30a vector (MilliporeSigma, Burlington, Massachusetts, USA). The nucleotide sequences were verified experimentally.

The two DARPins and the MBP-DUSP1 CD (Cys258Ser) fusion protein were expressed in *E. coli* strain BL21-CodonPlus (DE3)-RIL (Agilent Technologies, Santa Clara, California, USA). Cultures were grown to mid-log phase (OD_600_ = 0.4–0.6) at 37°C in Luria broth supplemented with 100 µg ml^−1^ ampicillin, 30 µg ml^−1^ chloramphenicol and 0.2% glucose to produce the MBP-DUSP1 CD fusion protein, while 35 µg ml^−1^ kanamycin was used instead of ampicillin to produce the DARPins. Proteins were induced with isopropyl β-d-1-thiogalactopyranoside (IPTG) at a concentration of 1 m*M* for 4 h at 37°C and 250 rev min^−1^. The cells were harvested by centrifugation and stored at −80°C until further use.

Protein purification was carried out at 4°C using a Bio-Rad NGC Chromatography System with pre-packed chromatography columns (GE Healthcare Biosciences, Marlborough, Massachusetts, USA) as described previously (Gumpena *et al.*, 2018 ▸). To prepare complexes of the DARPins with the maltose-bound form of MBP (in its ‘closed’ conformation), d-(+)-maltose monohydrate (maltose) solution was added to the purified MBP fusion protein–DARPin complexes at a final concentration of 1 m*M*.

### 2.2. Crystallization and data collection   

Complexes of MBP-DUSP1 CD with DARPin off7 (31 mg ml^−1^) and DARPin 16 (27 mg ml^−1^) in the presence and absence of maltose were screened for initial crystallization conditions at 19°C using a Griffin crystallization robot (Art Robbins Instruments, Sunnyvale, California, USA) and commercially available screens. Crystals suitable for data collection were obtained after further optimization of the initial hits in a grid screen using a 15-well EasyXtal Tool (Qiagen, Germantown, Maryland, USA). Crystallization conditions and cryosolutions are summarized in Table 2 ▸. For the complex of DARPin off7 with MBP-DUSP1 CD, the drop consisted of a 1:2 ratio of protein:well solution. A 1:1 protein:well solution ratio was used for the complexes of DARPin 16 with MBP-DUSP1 CD. Single crystals were retrieved, cryoprotected and immediately flash-cooled in liquid nitrogen.

Native X-ray diffraction data were collected for the MBP-DUSP1 CD–DARPin 16 complexes (with and without bound maltose) at −173°C using a MAR345 detector mounted on a Rigaku MicroMax-007 HF high-intensity microfocus generator equipped with VariMax HF optics (Rigaku Corporation, The Woodlands, Texas, USA) and operated at 40 kV and 30 mA. For the MBP-DUSP1 CD–DARPin off7 complex, the data were collected remotely on SER-CAT beamline 22-BM at the Advanced Photon Source, Argonne National Laboratory using a MAR 225 CCD detector. The data were integrated and scaled with *HKL*-3000 (Minor *et al.*, 2006 ▸). Data-collection statistics are reported in Table 3 ▸.

### 2.3. Structure solution and refinement   

All structures were solved by molecular replacement using *Phaser* from the *CCP*4 suite (McCoy *et al.*, 2007 ▸). Among the three complexes reported here, the structure of the DARPin off7 complex was solved first. A search model containing an ensemble of the following coordinates was employed as implemented in *Phaser*: MBP (from PDB entry 3h4z; Mueller *et al.*, 2010 ▸), DUSP4 CD (PDB entry 3ezz; Jeong *et al.*, 2009 ▸) and DARPin off7 (from PDB entry 1svx; Binz *et al.*, 2004 ▸). To solve the structure of DARPin 16 in complex with the open form of the MBP-DUSP1 CD fusion protein, the coordinates of MBP (from PDB entry 3h4z) and those of the DUSP1 CD and DARPin off7 complex from the previously determined structure were employed as search models. However, DARPin off7 is a three-repeat module (N3C), whereas DARPin 16 is a two-repeat module (N2C). Therefore, the extra repeat in DARPin off7 was deleted during the preparation of coordinates for molecular replacement. To solve the structure of DARPin 16 in complex with the closed (maltose-bound) form of the MBP-DUSP1 CD fusion protein, three ensembles were employed: those of the closed conformation of MBP (from PDB entry 3mp6; Bian *et al.*, 2011 ▸), along with those of the DUSP1 CD and those of DARPin 16, respectively, from the structure of the open form of MBP-DUSP1 CD in complex with DARPin 16. The models were rebuilt manually using *Coot* (Emsley & Cowtan, 2004 ▸) and refined with *phenix.refine* in the *PHENIX* software suite (Afonine *et al.*, 2012 ▸). Model validation was performed with *MolProbity* (Chen *et al.*, 2010 ▸). Ramachandran plots were prepared with *PROCHECK* (Laskowski *et al.*, 1993 ▸). Details of structure solution and refinement are summarized in Table 4 ▸. Representative electron density in the vicinity of the DUSP1 CD active site from the MBP-DUSP1 CD–DARPin 16 structure in the absence of maltose is shown in Supplementary Fig. S1.

### 2.4. Analysis of the structures   

All alignments of experimental structures to calculate r.m.s.d. values were performed in *PyMOL* (v.1.8; Schrödinger). Figures were also prepared with *PyMOL*. The interface areas were calculated with the *PDBePISA* server (Krissinel & Henrick, 2007 ▸) and the interfaces were further analyzed with *LigPlot* ^+^ (Laskowski & Swindells, 2011 ▸).

## 3. Results   

### 3.1. DARPins off7 and 16 facilitate crystallization of the MBP-DUSP1 CD fusion protein   

The maltose-free or ‘open’ form of the MBP-DUSP1 CD fusion protein was crystallized in complex with DARPin off7, while both the maltose-free and maltose-bound or ‘closed’ forms of the fusion protein were crystallized in complex with DARPin 16, yielding a total of three different crystal structures. Multiple ‘hits’ were obtained from commercial crystal screens for all three samples. The crystals of the DARPin 16 complexes contain one heterodimer per asymmetric unit in both crystal forms, whereas the crystals of MBP-DUSP1 CD in complex with DARPin off7 contain two heterodimers per asymmetric unit that are related by twofold noncrystallographic symmetry. The three crystal forms reported in this work (Table 1 ▸), along with the previously reported complex with the monobody (PDB entry 6apx), represent five crystallographically independent copies of the DUSP1 CD. When the C^α^ coordinates of the DUSP1 CD molecules in the three crystal forms reported here are superimposed with those of the DUSP1 CD in PDB entry 6apx, the root-mean-square deviation (r.m.s.d.) among the aligned structures varies between 0.26 and 0.32 Å, which is within the limit of the coordinate error for the resolution at which the structures were solved. This indicates that the presence of MBP and the MBP-binding proteins in the crystal lattice did not distort the structure of the DUSP1 CD.

In each of the structures, interactions between different molecular entities (MBP, the DUSP1 CD and the MBP-binding protein) exist and are integral components of the crystal lattices. However, the crystal-packing interactions are different in each structure and there are no noteworthy similarities between them, except that the DARPins exhibit a tendency to contact adjacent DUSP1 CD molecules *via* their N- and C-terminal caps (Supplementary Table S1). It is not possible to predict, therefore, which MBP-binding protein will be the most effective at promoting the crystallization of a particular MBP fusion protein. Instead, this must be determined empirically. Nevertheless, the fact that it was possible to obtain several different structures of an MBP fusion protein that was recalcitrant to crystallization by using this approach validates its utility.

**Figure 1:**
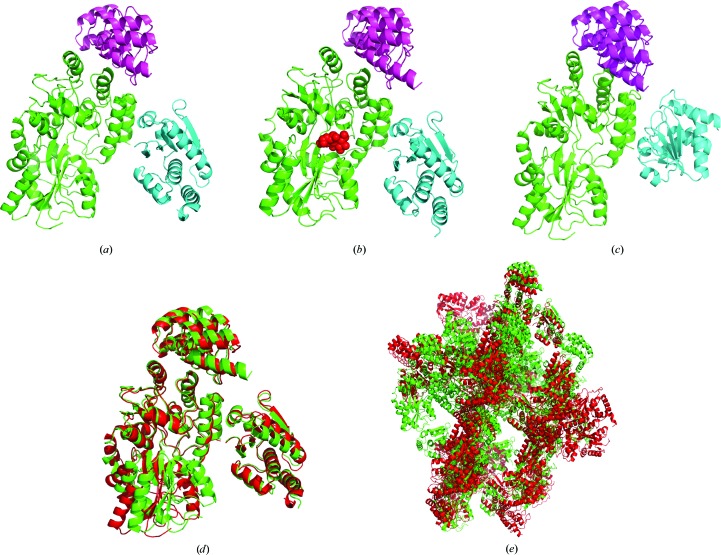
Conformations of the MBP-DUSP1 CD–DARPin complexes. The MBP, DUSP1 CD and DARPin moieties are depicted in green, cyan and magenta, respectively, in (*a*)–(*c*). MBP has the same orientation in all panels. (*a*) The DARPin 16 complex in the absence of bound maltose. (*b*) The DARPin 16 complex with bound maltose. Maltose is shown as red spheres. (*c*) The off7 complex. (*d*) Structural alignment of the two DARPin 16 complexes with (green) and without (red) maltose. (*e*) Crystal packing of DARPin 16 complexes with (green) and without (red) maltose.

The structures of the three MBP-DUSP1 CD–DARPin complexes are shown in Figs. 1 ▸(*a*), 1 ▸(*b*) and 1 ▸(*c*), with the MBP entities viewed from the same perspective. Relative to the conformation of MBP, the orientation of the DUSP1 CD in the DARPin off7 complex is very different to that in the DARPin 16 complexes. Interestingly, the two DARPin 16 complexes (in the presence and absence of maltose) align remarkably well (Fig. 1 ▸ *d*). The main difference between them is a shift in the position of one lobe of the MBP structure that is triggered by the binding of maltose. The crystal packing of the two DARPin 16 complexes is not the same however (Fig. 1 ▸ *e*).

### 3.2. Potential impact of surface-entropy-reduction mutations in MBP   

The MBP employed in this study has several surface-entropy-reduction mutations (Asp82Ala/Lys83Ala/Glu172Ala/Asn173Ala/Lys239Ala) that are designed to facilitate the crystallization of MBP fusion proteins (Moon *et al.*, 2010 ▸). None of these mutations overlap with the binding sites for DARPins off7 or 16. In all of the structures reported here at least one of the SER mutations mentioned above is involved in crystal contacts (Supplementary Table S2) with the neighboring molecules. Crystal contacts are 4.4 Å apart or closer (Carugo & Argos, 1997 ▸). In the MBP-DUSP1 CD–DARPin off7 complex, the side chain of Ala83 in MBP is projected into the hydrophobic pocket formed by Asn124, Pro126, Glu131 and Leu135 in a symmetry-related MBP. The presence of a much larger and charged lysine side chain in the place of Ala83, as exists in wild-type MBP, would have negatively influenced the crystal packing. Hence, it is possible that, along with the DARPins, the surface-entropy-reduction mutations helped to promote crystallization of the protein complexes.

### 3.3. DARPins 16 and off7 bind to the same site on the surface of MBP in a remarkably similar manner   

**Figure 2:**
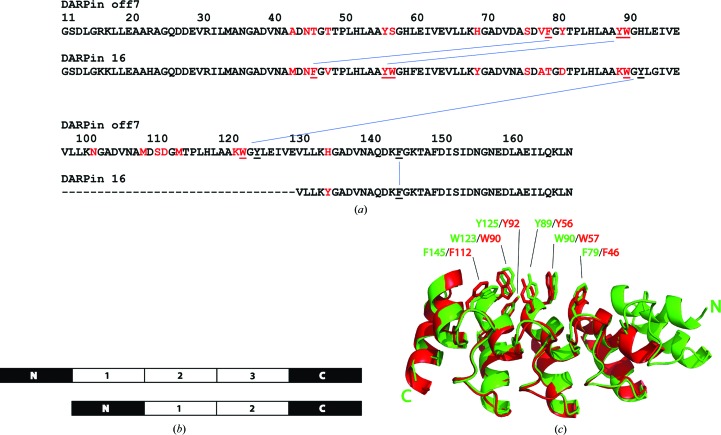
Similarities between binding paratopes in DARPins off7 and 16. (*a*) Amino-acid sequence alignment between DARPins off7 and 16. The hyphens denote the absence of a third designed repeat in DARPin 16. Randomized interaction residues are colored red. The six amino acids that form the conserved core of the binding paratope are underlined and their corresponding positions in the two amino-acid sequences are indicated by blue lines. (*b*) Schematic view of the structural alignment between DARPins off7 and 16, illustrating the incongruity of the N-terminal capping and designed repeats. (*c*) Ribbon representation of the structural alignment between DARPins off7 (green) and 16 (red). The six conserved residues that form the core of the binding paratopes at the interface with MBP are shown as sticks.

Although the interaction between DARPin off7 and MBP has been described in detail (Binz *et al.*, 2004 ▸), until now it had been unknown where and how DARPin 16 binds to MBP. The crystal structures described in this report reveal that the binding sites for the two DARPins overlap extensively. Not only that, but the key residues that comprise the core of the binding paratopes on the two DARPins are identical. Remarkably, however, these conserved residues do not align with one another in the amino-acid sequences of DARPins off7 and 16 (Fig. 2 ▸ *a*) and so their equivalence originally went unrecognized. Both DARPins have the same N- and C-terminal capping repeats, but DARPin 16 has only two designed repeats whereas off7 has three (Figs. 2 ▸ *a* and 2 ▸ *b*). However, when the structure of the DARPin off7–MBP complex (PDB entry 1svx) is superimposed with the coordinates of DARPin 16 bound to MBP, the structural alignment juxtaposes the two DARPins in such a manner that the N-terminal capping repeat of off7 has no counterpart in DARPin 16 (Fig. 2 ▸ *b*). As shown in Figs. 2 ▸(*a*) and 2 ▸(*c*), even though they originate from different locations in their respective amino-acid sequences, the six key MBP-binding residues in the two DARPins occupy virtually the same spatial positions. The interactions between these six key residues and MBP are predominantly hydrophobic in nature. There are additional interactions between each DARPin and MBP that are unique. A complete list of interacting residues is provided in Supplementary Tables S3 and S4.

## 4. Discussion   

The remarkable ability of MBP to enhance the solubility of its fusion partners and promote their crystallization has been well documented (Waugh, 2016 ▸). However, despite considerable effort it was not possible to obtain crystals of an MBP-DUSP1 CD fusion protein constructed for this purpose. We therefore decided to explore the potential utility of high-affinity MBP-binding proteins as co-crystallization ‘chaperones’ for MBP-DUSP1 CD. Remarkably, we readily obtained co-crystals of the MBP-DUSP1 CD fusion protein with the two different MBP-binding DARPins described here as well as with an MBP-binding monobody as described previously (Gumpena *et al.*, 2018 ▸). Complexes of the fusion protein with DARPin 16 crystallized in both the absence and presence of maltose, yielding a total of four unique structures at moderate resolution (2.2–2.5 Å). The DUSP fold is virtually identical in all of the structures of the DUSP1 CD, indicating that the presence of MBP and the MBP-binding proteins did not distort its structure.

In the work described here, the components of the DARPin–MBP-DUSP1 CD complexes were expressed separately and the lysates were mixed to form the complexes prior to purification. However, the routine use of MBP-binding co-crystallization chaperones would probably be facilitated by purifying a large quantity of the His-tagged DARPins (or monobody) and storing them for future use. Each new MBP fusion protein, following its purification, could then be mixed with a molar excess of the much smaller MBP-binding proteins and the complexes separated from the excess DARPins or monobody by size-exclusion chromatography.

The structure of MBP-DUSP1 CD in complex with DARPin 16 reveals for the first time how this DARPin interacts with MBP. Surprisingly, even though the amino acids in the designed regions of DARPin 16 and off7 appear to be dissimilar when their sequences are aligned, the two molecules bind to the same site on MBP and adopt remarkably similar poses. A structural alignment of the two DARPin–MBP complexes reveals six conserved residues that emanate from different locations in the amino-acid sequences of the DARPins yet occupy the same spatial positions at the interface with MBP. Hence, this binding paratope, which is primarily hydrophobic in nature, was obtained in two separate directed evolution experiments with DARPin scaffolds having two and three designed repeats, respectively (Binz *et al.*, 2004 ▸).

**Figure 3:**
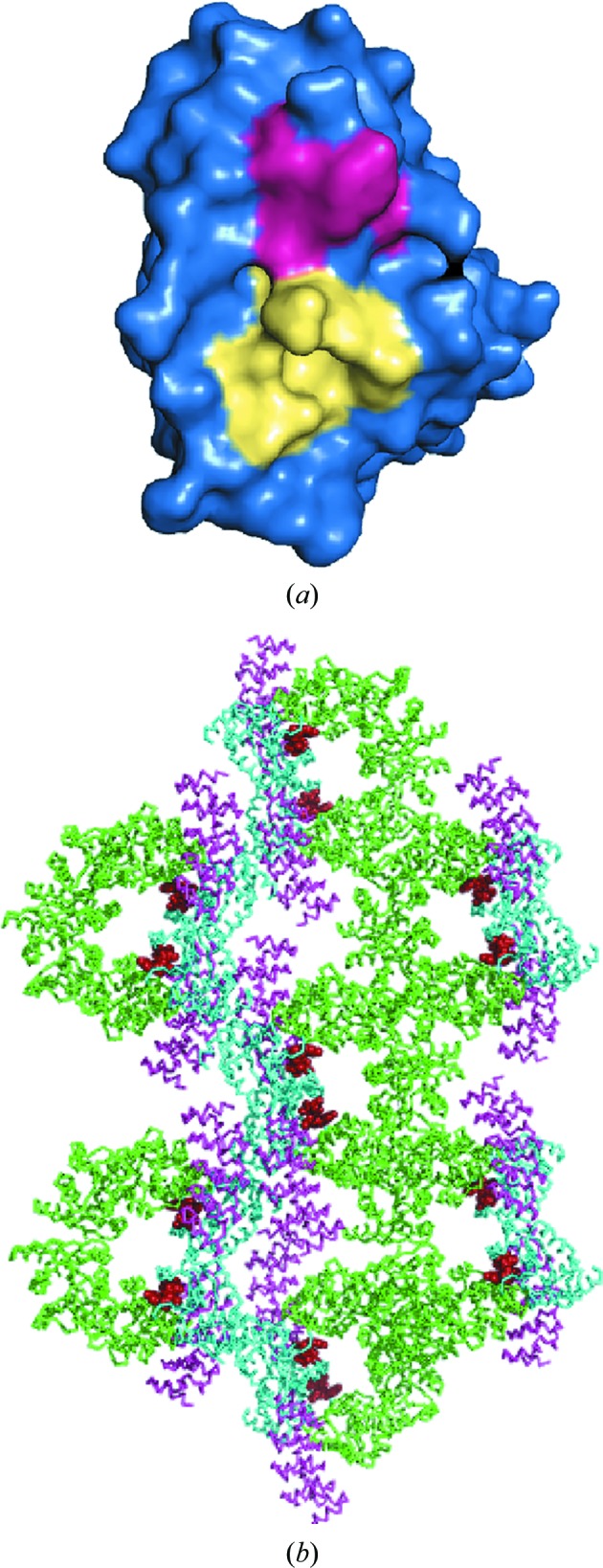
Solvent-accessibility of helix α1 in crystals of the MBP-DUSP1 CD–DARPin off7 complex. (*a*) Surface representation of the DUSP1 CD showing the location of helix α1 (pink) with respect to the active site (yellow). (*b*) Crystal packing of the MBP-DUSP1 CD–DARPin off7 complex. MBP, DARPin off 7 and DUSP1 CD residues are colored green, pink and cyan, respectively. Helix α1 residues are shown as red spheres.

Finally, we note that MBP fusion proteins have been used not only as tools to solve unknown structures but also to generate alternative crystal forms of a protein that may be more amenable to crystallographic fragment screening and structure-based drug design (Clifton *et al.*, 2015 ▸). The utilization of MBP-binding proteins as co-crystallization chaperones may have similar benefits. For example, the presence of a highly conserved active-site architecture in the DUSP family poses challenges for the development of specific inhibitors targeting their active sites alone (Bakan *et al.*, 2008 ▸). In a recent survey of DUSP structures, it was proposed that it may be possible to develop specific inhibitors of DUSP family members by exploiting the small variable surface features in the vicinity of their active sites (Jeong *et al.*, 2014 ▸). One such variable region in the DUSP1 CD that is located in close proximity to its active site is helix α1, which is formed by Ala186, Tyr187, His188, Ala189 and Ser190 (Fig. 3 ▸ *a*). In the crystal structure of MBP-DUSP1 CD with DARPin off7, helix α1 is positioned at the edge of a solvent channel with an inner diameter of approximately 21 Å and is readily accessible to small molecules, as is the active site (Fig. 3 ▸ *b*). On the other hand, in the crystal structures of the MBP-DUSP1 CD in complex with DARPin 16, helix α1 residues are occluded by a crystal contact with a neighboring molecule, rendering these crystal forms less advantageous for crystallographic fragment screening.

## 5. Conclusions   

Owing to its stochastic nature, there is a persistent demand for technological advances in the field of protein crystallization. For example, MBP has frequently been exploited to promote the crystallization of its fusion partners. However, despite considerable effort, we could not obtain crystals of an MBP-DUSP1 CD fusion protein by itself. For the first time, we have demonstrated that high-affinity MBP-binding DARPins can be employed to facilitate the crystallization of an MBP fusion protein. As proof of principle, we determined three different crystal structures of the MBP-DUSP1 CD fusion protein in complex with two different DARPins, one of which also included bound maltose. We propose that the DARPin technology reported here can generally improve the probability of obtaining crystals of an MBP fusion protein.

## Supplementary Material

PDB reference: MBP-DUSP1 CD in complex with DARPin off7, 6d65

PDB reference: MBP-DUSP1 CD in complex with DARPin 16, 6d66

PDB reference: 6d67

Supplementary Tables and Figure.. DOI: 10.1107/S2053230X18009901/va5017sup1.pdf

## Figures and Tables

**Table 1:** Macromolecule-production information

DARPin off7	
Source	Synthetic construct
DNA source	Plasmid ID 1514_i, Department of Biochemistry, University of Zurich, Switzerland
Forward primer	GGGAATTCCATATGAGAGGATCGCATCACCATCA (PE-2888)
Reverse primer	CCGCTCGAGTTAATTAAGCTTTTGCAGGATTTCCGC (PE-2889)
Cloning vector	pET-30a
Expression vector	pRG2743
Expression host	*E. coli* BL21-CodonPlus (DE3)-RIL
Complete amino-acid sequence of the construct produced	MRGSHHHHHHGSDLGRKLLEAARAGQDDEVRILMANGADVNAADNTGTTPLHLAAYSGHLEIVEVLLKHGADVDASDVFGYTPLHLAAYWGHLEIVEVLLKNGADVNAMDSDGMTPLHLAAKWGYLEIVEVLLKHGADVNAQDKFGKTAFDISIDNGNEDLAEILQKLN
DARPin 16	
Source	Synthetic construct
DNA source	Plasmid ID 496_i, Department of Biochemistry, University of Zurich, Switzerland
Forward primer	GGGAATTCCATATGAGAGGATCGCATCACCATCA (PE-2888)
Reverse primer	CCGCTCGAGTTAATTAAGCTTTTGCAGGATTTCCGC (PE-2889)
Cloning vector	pET-30a
Expression vector	pRG2744
Expression host	*E. coli* BL21-CodonPlus (DE3)-RIL
Complete amino-acid sequence of the construct produced	MRGSHHHHHHGSDLGKKLLEAARAGQDDEVRILMANGADVNADDTEGNTPLHLVAVHGHLEIVEVLLKYGADVNAHDVWGQTPLHLAAYYDHLEIVEVLLKYGADVNADDDTGITPLHLAARWGHLEIVEVLLKYGADVNAQDKFGKTAFDISIDNGNEDLAEILQKLN

**Table 2:** Crystallization

	MBP-DUSP1 CD in complex with DARPin off7	MBP-DUSP1 CD in complex with DARPin 16 (MBP open form)	MBP-DUSP1 CD in complex with DARPin 16 (MBP closed form)
Method	Vapor diffusion, hanging drop	Vapor diffusion, hanging drop	Vapor diffusion, hanging drop
Plate type	EasyXtal 15-well plate (Qiagen)	EasyXtal 15-well plate (Qiagen)	EasyXtal 15-well plate (Qiagen)
Temperature (K)	292	292	292
Protein concentration (mg ml^−1^)	31	27	27
Composition of reservoir solution	0.15 *M* sodium chloride, 0.1 *M* sodium cacodylate pH 6.0, 2.0 *M* ammonium sulfate	0.02 *M* DL-glutamic acid, 0.02 *M* DL-alanine, 0.02 *M* glycine, 0.02 *M* DL-lysine, 0.02 *M* DL-serine, 0.1 *M* Tris–Bicine pH 8.5, 25%(*v*/*v*) MPD, 25%(*v*/*v*) PEG 1000, 25%(*v*/*v*) PEG 3350	0.02 *M* DL-glutamic acid, 0.02 *M* DL-alanine, 0.02 *M* glycine, 0.02 *M* DL-lysine, 0.02 *M* DL-serine, 0.1 *M* Tris–Bicine pH 8.5, 25%(*v*/*v*) MPD, 25%(*w*/*v*) PEG 1000, 25%(*w*/*v*) PEG 3350
Volume and ratio of drop	3 µl, 1:2 mixture of protein and reservoir solutions	3 µl, 1:1 mixture of protein and reservoir solutions	3 µl, 1:1 mixture of protein and reservoir solutions
Volume of reservoir (ml)	0.5	0.5	0.5
Cryosolution	20%(*v*/*v*) ethanol in mother liquor	2%(*w*/*v*) xylitol in mother liquor	Paraffin oil
Soaking time in cryosolution	12 h	16 h	5 s

**Table 3:** Data collection and processing Values in parentheses are for the highest resolution shell.

	MBP-DUSP1 CD in complex with DARPin off7	MBP-DUSP1 CD in complex with DARPin 16 (MBP open form)	MBP-DUSP1 CD in complex with DARPin 16 (MBP closed form)
Diffraction source	22-BM, SER-CAT	MicroMax-007 HF	MicroMax-007 HF
Wavelength (Å)	1.0	1.5418	1.5418
Temperature (K)	100	100	100
Detector	MAR 225 CCD	MAR345 image plate	MAR345 image plate
Crystal-to-detector distance (mm)	275	200	175
Rotation range per image (°)	1.0	0.25	0.5
Total rotation range (°)	180	180	180
Exposure per image (s)	0.5	300	600
Space group	*P*2_1_2_1_2_1_	*P*4_1_2_1_2	*P*2_1_2_1_2_1_
Unit-cell parameters			
*a* (Å)	75.3	79.9	75.2
*b* (Å)	109.5	79.9	84.6
*c* (Å)	218.5	265.7	105.9
α = β = γ (°)	90	90	90
Mosaicity (°)	0.47	0.69	0.30
Resolution range (Å)	50.00–2.34 (2.38–2.34)	50.00–2.22 (2.30–2.22)	50.00–2.55 (2.64–2.55)
Total No. of reflections	372374	461040	146915
No. of unique reflections	75855 (3726)	43247 (3697)	22649 (2139)
Completeness (%)	99.8 (100)	98.6 (86.5)	99.6 (96.4)
Multiplicity	4.9 (4.6)	10.7 (7.7)	6.5 (3.4)
Mean *I*/σ(*I*)	17.1 (2.3)	46.4 (3.6)	18.1 (2.0)
*R*_merge_	0.117 (0.763)	0.050 (0.388)	0.100 (0.603)
Overall *B* factor from Wilson plot (Å^2^)	32.0	27.0	49.8
No. of heterodimers in the asymmetric unit	2	1	1

**Table 4:** Structure solution and refinement Values in parentheses are for the highest resolution shell.

	MBP-DUSP1 CD in complex with DARPin off7	MBP-DUSP1 CD in complex with DARPin 16 (MBP open form)	MBP-DUSP1 CD in complex with DARPin 16 (MBP closed form)
Resolution range (Å)	38.67–2.34 (2.37–2.34)	36.44–2.22 (2.27–2.22)	38.57–2.55 (2.66–2.55)
Completeness (%)	99.3	99.3	99.3
No. of reflections, working set	75783 (2250)	43109 (2439)	22558 (2531)
No. of reflections, test set	3728 (121)	2135 (138)	1098 (140)
Final *R*_cryst_	0.186	0.154	0.183
Final *R*_free_	0.232	0.189	0.258
No. of non-H atoms			
Protein			
MBP	5640	2838	2805
DUSP1 CD	2254	1194	1134
DARPin	2335	951	960
Ligands			
Maltose			23
Sulfate	115		
Phosphate		5	5
Ethanol	63		
Glycerol	36		
PEG		21	7
PG4		13	
PGE		30	
Glycine		15	
D-Alanine		6	
Ethane-1,2-diol		48	12
Water	611	307	81
Average *B* factors (Å^2^)			
Protein			
MBP	37.1	32.6	48.0
DUSP1 CD	34.9	30.9	51.2
DARPin	39.1	29.3	50.2
Ligands			
Maltose			43.5
Sulfate	56.1		
Phosphate		22.0	43.1
Ethanol	49.3		
Glycerol	56.0		
PEG		57.8	65.1
PG4		53.8	
PGE		51.1	
Glycine		56.5	
D-Alanine		51.6	
Ethane-1,2-diol		53.0	67.5
Water	38.4	37.5	49.9
R.m.s.d. from ideal geometry			
Bond lengths (Å)	0.008	0.007	0.008
Bond angles (°)	0.9	0.8	0.9
Ramachandran plot			
Favored regions (%)	91.5	93.3	90.0
Allowed regions (%)	8.3	6.4	9.4
Generously allowed region (%)	0	0.2	0.4
Outliers (%)	0.2	0	0.2
PDB code	6d65	6d66	6d67

## References

[bb1] Abola, E., Kuhn, P., Earnest, T. & Stevens, R. C. (2000). *Nature Struct. Biol.* **7**, 973–977. 10.1038/80754 11104004

[bb2] Afonine, P. V., Grosse-Kunstleve, R. W., Echols, N., Headd, J. J., Moriarty, N. W., Mustyakimov, M., Terwilliger, T. C., Urzhumtsev, A., Zwart, P. H. & Adams, P. D. (2012). *Acta Cryst.* D**68**, 352–367. 10.1107/S0907444912001308 PMC3322595 22505256

[bb3] Bakan, A., Lazo, J. S., Wipf, P., Brummond, K. M. & Bahar, I. (2008). *Curr. Med. Chem.* **15**, 2536–2544. 10.2174/092986708785909003 PMC2764859 18855677

[bb4] Bell, M. R., Engleka, M. J., Malik, A. & Strickler, J. E. (2013). *Protein Sci.* **22**, 1466–1477. 10.1002/pro.2356 PMC3831663 24038604

[bb5] Bian, C. *et al.* (2011). *EMBO J.* **30**, 2829–2842. 10.1038/emboj.2011.193 PMC3160252 21685874

[bb6] Binz, H. K., Amstutz, P., Kohl, A., Stumpp, M. T., Briand, C., Forrer, P., Grütter, M. G. & Plückthun, A. (2004). *Nature Biotechnol.* **22**, 575–582. 10.1038/nbt962 15097997

[bb7] Carugo, O. & Argos, P. (1997). *Protein Sci.* **6**, 2261–2263. 10.1002/pro.5560061021 PMC2143556 9336849

[bb8] Chen, V. B., Arendall, W. B., Headd, J. J., Keedy, D. A., Immormino, R. M., Kapral, G. J., Murray, L. W., Richardson, J. S. & Richardson, D. C. (2010). *Acta Cryst.* D**66**, 12–21. 10.1107/S0907444909042073 PMC2803126 20057044

[bb9] Clifton, M. C. *et al.* (2015). *PLoS One*, **10**, e0125010. 10.1371/journal.pone.0125010 PMC4409056 25909780

[bb10] Cooper, D. R., Boczek, T., Grelewska, K., Pinkowska, M., Sikorska, M., Zawadzki, M. & Derewenda, Z. (2007). *Acta Cryst.* D**63**, 636–645. 10.1107/S0907444907010931 17452789

[bb11] Dale, G. E., Oefner, C. & D’Arcy, A. (2003). *J. Struct. Biol.* **142**, 88–97. 10.1016/s1047-8477(03)00041-8 12718922

[bb12] Emsley, P. & Cowtan, K. (2004). *Acta Cryst.* D**60**, 2126–2132. 10.1107/S0907444904019158 15572765

[bb13] Gilbreth, R. N., Esaki, K., Koide, A., Sidhu, S. S. & Koide, S. (2008). *J. Mol. Biol.* **381**, 407–418. 10.1016/j.jmb.2008.06.014 PMC2582520 18602117

[bb14] Gumpena, R., Lountos, G. T., Raran-Kurussi, S., Tropea, J. E., Cherry, S. & Waugh, D. S. (2018). *Protein Sci.* **27**, 561–567. 10.1002/pro.3328 PMC5775162 29052270

[bb15] Jeong, D. G., Jung, S.-K., Yoon, T.-S., Woo, E.-J., Kim, J. H., Park, B. C., Ryu, S. E. & Kim, S. J. (2009). *Proteins*, **76**, 763–767. 10.1002/prot.22423 19415758

[bb16] Jeong, D. G., Wei, C. H., Ku, B., Jeon, T. J., Chien, P. N., Kim, J. K., Park, S. Y., Hwang, H. S., Ryu, S. Y., Park, H., Kim, D. S., Kim, S. J. & Ryu, S. E. (2014). *Acta Cryst.* D**70**, 421–435. 10.1107/S1399004713029866 24531476

[bb17] Jin, T., Chuenchor, W., Jiang, J., Cheng, J., Li, Y., Fang, K., Huang, M., Smith, P. & Xiao, T. S. (2017). *Sci. Rep.* **7**, 40991. 10.1038/srep40991 PMC5256280 28112203

[bb18] Krissinel, E. & Henrick, K. (2007). *J. Mol. Biol.* **372**, 774–797. 10.1016/j.jmb.2007.05.022 17681537

[bb19] Laskowski, R. A., MacArthur, M. W., Moss, D. S. & Thornton, J. M. (1993). *J. Appl. Cryst.* **26**, 283–291.

[bb20] Laskowski, R. A. & Swindells, M. B. (2011). *J. Chem. Inf. Model.* **51**, 2778–2786. 10.1021/ci200227u 21919503

[bb21] Longenecker, K. L., Garrard, S. M., Sheffield, P. J. & Derewenda, Z. S. (2001). *Acta Cryst.* D**57**, 679–688. 10.1107/s0907444901003122 11320308

[bb22] McCoy, A. J., Grosse-Kunstleve, R. W., Adams, P. D., Winn, M. D., Storoni, L. C. & Read, R. J. (2007). *J. Appl. Cryst.* **40**, 658–674. 10.1107/S0021889807021206 PMC2483472 19461840

[bb23] Minor, W., Cymborowski, M., Otwinowski, Z. & Chruszcz, M. (2006). *Acta Cryst.* D**62**, 859–866. 10.1107/S0907444906019949 16855301

[bb24] Moon, A. F., Mueller, G. A., Zhong, X. & Pedersen, L. C. (2010). *Protein Sci.* **19**, 901–913. 10.1002/pro.368 PMC2868234 20196072

[bb25] Mueller, G. A., Edwards, L. L., Aloor, J. J., Fessler, M. B., Glesner, J., Pomés, A., Chapman, M. D., London, R. E. & Pedersen, L. C. (2010). *J. Allergy Clin. Immunol.* **125**, 909–917.e4. 10.1016/j.jaci.2009.12.016 PMC2885876 20226507

[bb26] Ruggiero, A., Smaldone, G., Squeglia, F. & Berisio, R. (2012). *Protein Pept. Lett.* **19**, 732–742. 10.2174/092986612800793172 22489782

[bb27] Slack, D. N., Seternes, O. M., Gabrielsen, M. & Keyse, S. M. (2001). *J. Biol. Chem.* **276**, 16491–16500. 10.1074/jbc.M010966200 11278799

[bb28] Waugh, D. S. (2016). *Protein Sci.* **25**, 559–571. 10.1002/pro.2863 PMC4815407 26682969

