# Supplementary material for: MBP-binding DARPins facilitate the crystallization of an MBP fusion protein
Source: Acta Crystallogr F Struct Biol Commun. 2018 Aug 29;74(Pt 9):549–57. doi: 10.1107/S2053230X18009901 (PMC6130421; doi:10.1107/S2053230X18009901)
Supplement: Supplementary file 1 [file f-74-00549-sup1.pdf]

STRUCTURAL BIOLOGY COMMUNICATIONS

**Volume 74 (2018)**

**Supporting information for article:**

# MBP-binding DARPins facilitate the crystallization of an MBP fusion protein

**Rajesh Gumpena, George T. Lountos and David S. Waugh**

**Table S1** Interactions between the DARPins and the DUSP1-CD molecules

| **Complex** | **DARPin region (*symmetry*)** | **Residues involved in the interaction** | **Secondary structure element of the DUSP1-CD** | **Residues involved in the interaction** | **Interface area (Å$^2$)** |
|---|---|---|---|---|---|
| MBP-DUSP1-CD with DARPin off7 | N-terminal cap (*x, y, z*) | Leu14, Glu29, Ile32, Leu33, Ala35, Asn36[§] | β3-β4 loop | Glu215[§], Gly216, His217, Tyr218[§] | 290.4[a] |
| | | | strand β4 | Gln219, Tyr220[§] | |
| MBP-DUSP1-CD with DARPin 16 (MBP open form) | C-terminal cap (*-x-3/2, y-1/2, -z+1/4*) | Ile121, Asp122[§], Gly124, Glu126*, Glu130[¶], Gln133 | strand β2 | Ser185 | 419.7 |
| | | | helix α1 | Tyr187, His188 | |
| | | | β5-α5 loop | Gln259[§], Ala260 | |
| | | | α5-α6 loop | Arg292* Ser293[¶], Ile294 | |
| MBP-DUSP1-CD with DARPin 16 | C-terminal cap | Phe117, Ile121, | helix α1 | Tyr187, His188 | 358.2 |

| | | | | | |
|---|---|---|---|---|---|
| (MBP closed form) | (*x-1/2, -y+1/2, -z*) | Asp122[§], Asn123, Gly124, Glu126[¶], Glu130, Gln133* | β5-α5 loop | Gln259[§], Ala260, Ile262 | |
| | | | α5-α6 loop | Arg292[¶], Ile294* | |
| | N-terminal cap (*-x, y-1/2, -z-1/2*) | His8[§], Gly11, Ala35, Asn36, Gly37, Asp39, Asn41* | β4-α3 loop | His229, Lys230 | 363.7 |
| | | | α5-α6 loop | Ser296, Pro297[§] | |
| | | | helix α6 | Phe299, Gly303, Gln307* | |

[§], [¶], * are the corresponding residues involved in hydrogen bonding.

A cut-off 3.3 Å for hydrogen bonding and 3.9 Å for hydrophobic interactions was applied.

[a] Average interface area in the heterodimer.

**Table S2** Residues involved in crystal contacts mediated by the SER mutations of the MBP

A cut-off 4.4 Å is employed for the crystal contacts.

| | Complex of the MBP-DUSP1-CD with DARPin | | | |
|---|---|---|---|---|
| SER mutation on MBP | Off7 | | 16 (MBP open form) | 16 (MBP closed form) |
| | Chain A | Chain C | | |
| Asp82Ala | Tyr242[a] | Glu131[a] | Met34[b]<br>Ala35[b]<br>Gly37[b] | Lys101[b] |
| Lys83Ala | | Asn124[a]<br>Pro126[a]<br>Glu131[a]<br>Leu135[a] | Tyr69[b] | |
| Glu172Ala | | | | |
| Asn173Ala | | | | |
| Lys239Ala | Ala84[a] | | Gln307[c] | |

[a] Residues from the symmetry related MBP.

[b] Residues from the symmetry related DARPin.

[c] Residues from the symmetry related DUSP1-CD.

**Table S3** Hydrogen bonds and hydrophobic interactions

A cut-off 3.3 Å for hydrogen bonding and 3.9 Å for hydrophobic interactions was applied.

(*a*) List of the H-bonds and hydrophobic interactions between MBP and DARPin in the MBP-DUSP1-CD/off7 complex

| DARPin off7 designed repeat module number | DARPin off7 (chain-B) interaction residue | H-bond (Å) | MBP interaction residue (chain-A) | DARPin off7 (chain-D) interaction residue | H-bond (Å) | MBP interaction residue (chain-C) |
|---|---|---|---|---|---|---|
| 1 | | | | | | |
| | Tyr56 | 3.0 | Lys140 | Tyr56 | 2.8 | Lys140 |
| | Tyr56 | | Lys202 | Tyr56 | | Lys202 |
| | | | | | | |
| 2 | Val78 | | Ser352 | Val78 | | Ser352 |
| | Val78 | | Gly353 | Val78 | | Gly353 |
| | Phe79 | | Val196 | Phe79 | | Val196 |
| | Phe79 | | Lys200 | Phe79 | | Lys200 |
| | Phe79 | | Ala351 | Phe79 | | Ala351 |
| | Phe79 | | Gly353 | Phe79 | | Gly353 |
| | Tyr81 | | Lys200 | | | |
| | Tyr81 | 2.6 | Asn201 | Tyr81 | 2.4 | Asn201 |
| | | | | | | |
| | Tyr89 | 2.8 | Lys137 | Tyr89 | 2.6 | Lys137 |
| | Tyr89 | | Asn201 | | | |

| | | | | | | |
|---|---|---|---|---|---|---|
| | Tyr89 | 2.6 | His203 | Tyr89 | 2.6 | His203 |
| | Trp90 | | Asp136 | Trp90 | | Asp136 |
| | | | | Trp90 | | Lys137 |
| | Trp90 | | Lys140 | | | |
| | Trp90 | 2.9 | Asn201 | Trp90 | 3.0 | Asn201 |
| | Trp90 | | His203 | Trp90 | | His203 |
| | | | | | | |
| | | | | | | |
| | Asp112 | | Lys200 | | | |
| **3** | | | | | | |
| | Trp123 | | Lys137 | Trp123 | | Lys137 |
| | Tyr125 | 2.6 | Lys137 | Tyr125 | 2.6 | Lys137 |

(*b*) List of the H-bonds and hydrophobic interactions between MBP and DARPin in the MBP-DUSP1-CD/DARPin 16 complexes

| **DARPin16 designed repeat module number** | **DARPin 16 (open form of MBP) interaction residue** | **H-bond (Å)** | **MBP interaction residue** | **DARPin 16 (closed form of MBP) interaction residue** | **H-bond (Å)** | **MBP interaction residue** |
|---|---|---|---|---|---|---|
| | His23 | | Lys202 | His23 | | Lys202 |
| | | | | | | |
| | Asn45 | | Ser352 | Asn45 | | Ser352 |
| **1** | Phe46 | | Val196 | Phe46 | | Val196 |
| | Phe46 | | Lys200 | Phe46 | | Lys200 |

| | | | | | | |
|---|---|---|---|---|---|---|
| | Phe46 | | Ala350 | Phe46 | | Ala350 |
| | Phe46 | | Ala351 | Phe46 | | Ala351 |
| | Phe46 | | Gly353 | Phe46 | | Gly353 |
| | Val48 | | Lys200 | Val48 | | Lys200 |
| | Leu53 | | Lys202 | | | |
| | Tyr56 | | Pro133 | Tyr56 | | Pro133 |
| | | | | Tyr56 | | Asp136 |
| | Tyr56 | 2.9 | Lys137 | Tyr56 | 2.6 | Lys137 |
| | Tyr56 | | Asn201 | Tyr56 | | Asn201 |
| | Tyr56 | 2.6 | His203 | Tyr56 | 2.6 | His203 |
| | Trp57 | | Asp136 | Trp57 | | Asp136 |
| | Trp57 | | Lys137 | | | |
| | Trp57 | 3.0 | Asn201 | Trp57 | | Asn201 |
| | Trp57 | | His203 | Trp57 | | His203 |
| | | | | | | |
| | Asp77 | 3.0 | Lys200 | Asp77 | | Lys200 |
| | Thr79 | | Lys200 | Thr79 | | Asp197 |
| | Asp81 | 2.5 | Lys200 | Asp81 | 2.9 | Lys200 |
| **2** | Trp90 | | Pro133 | | | |
| | Trp90 | | Ala134 | Trp90 | | Ala134 |
| | Trp90 | | Lys137 | Trp90 | | Lys137 |
| | Tyr92 | 2.5 | Lys137 | Tyr92 | 2.6 | Lys137 |
| | | | | | | |
| | Lys111 | | Thr193 | Lys111 | | Thr193 |
| | Lys111 | 3.2 | Asp197 | Lys111 | 2.4 | Asp197 |

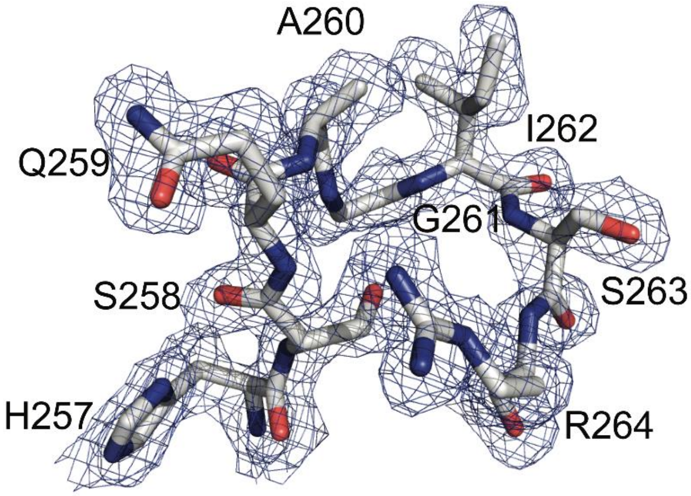


**Figure S1** The residues forming a portion of the active site of DUSP1 from the MBP-DUSP1-CD complex with DARpin16 (MBP open form) superimposed onto the final $2F_o$-$F_c$ electron density map (blue) contoured at 1σ level (2.22 Å resolution).
